# Cost-effectiveness analysis for HbA1c test intervals to screen patients with type 2 diabetes based on risk stratification

**DOI:** 10.1186/s12902-021-00771-0

**Published:** 2021-05-22

**Authors:** Sachiko Ohde, Kensuke Moriwaki, Osamu Takahashi

**Affiliations:** 1 Graduate School of Public Health, Clinical Epidemiology and HTA Center St. Luke’s International University, 3-6-2 Akashi-cho, Chuo, Tokyo, 104-0044 Japan; 2 Comprehensive Unit for Health Economic Evidence Review and Decision Support (CHEERS), Research Organization of Science and Technology, Ritsumeikan University, #209, Research Park Bid. No. 2, 134, Minami-machi, Chudoji, Simogyo-ku,, Kyoto, 600-8813 Japan

**Keywords:** Type 2 diabetes mellitus screening, HbA1c measurement, Cost effectiveness analysis

## Abstract

**Background:**

The best HbA1c test interval strategy for detecting new type 2 diabetes mellitus (T2DM) cases in healthy individuals should be determined with consideration of HbA1c test characteristics, risk stratification towards T2DM and cost effectiveness.

**Methods:**

State transition models were constructed to investigate the optimal screening interval for new cases of T2DM among each age- and BMI-stratified health individuals. Age was stratified into 30–44-, 45–59-, and 60–74-year-old age groups, and BMI was also stratified into underweight, normal, overweight and obesity. In each model, different HbA1c test intervals were evaluated with respect to the incremental cost-effectiveness ratio (ICER) and costs per quality-adjusted life year (QALY). Annual intervals (Japanese current strategy), every 3 years (recommendations in US and UK) and intervals which are tailored to each risk stratification group were compared. All model parameters, including costs for screening and treatment, rates for complications and mortality and utilities, were taken from published studies. The willingness-to-pay threshold in the cost-effectiveness analysis was set to US $50,000/QALY.

**Results:**

The HbA1c test interval for detecting T2DM in healthy individuals varies by age and BMI. Three-year intervals were the most cost effective in obesity at all ages—30-44: $15,034/QALY, 45–59: $11,849/QALY, 60–74: $8685/QALY—compared with the other two interval strategies. The three-year interval was also the most cost effective in the 60–74-year-old age groups—underweight: $11,377/QALY, normal: $18,123/QALY, overweight: $12,537/QALY—and in the overweight 45–59-year-old group; $18,918/QALY. In other groups, the screening interval for detecting T2DM was found to be longer than 3 years, as previously reported. Annual screenings were dominated in many groups with low BMI and in younger age groups. Based on the probability distribution of the ICER, results were consistent among any groups.

**Conclusions:**

The three-year screening interval was optimal among elderly at all ages, the obesity at all ages and the overweight in 45–59-year-old group. For those sin the low-BMI and younger age groups, the optimal HbA1c test interval could be longer than 3 years. Annual screening to detect T2DM was not cost effective and should not be applied in any population.

**Supplementary Information:**

The online version contains supplementary material available at 10.1186/s12902-021-00771-0.

## Background

The number of patients with type 2 diabetes (T2DM) is expected to be close to 600 million worldwide by 2030. The HbA1c test to detect new cases of T2DM in a healthy population is one of the most effective ways to prevent and start early treatment for type 2 diabetes; however, the best frequency for HbA1c testing remains unclear. Although multiple studies have tried to investigate the optimal frequency for HbA1c testing, current guidelines about test frequencies to screen type 2 diabetes patients in a healthy population still rely on expert opinion.

Kahn et al. [1] suggested that 30- to 45-year-old people are required to have 3- to 5-year interval screening on the basis of cost effectiveness analysis. However, this study does not consider the possibility of an incidence rate for type 2 diabetes based on risk factors. Multiple studies have reported that subjects with obesity are more likely to have type 2 diabetes than people with a normal BMI [2–4]. In addition to body mass index level, age also matters in the development of type 2 diabetes. The prevalence of type 2 diabetes increases as people live longer, and the complication rate among older adults with type 2 diabetes is significantly higher for both acute and chronic microvascular and cardiovascular diseases than that among younger adults with type 2 diabetes. People with a higher risk of onset of type 2 diabetes seem to require screening at shorter intervals than those with a lower risk [5, 6]. Previously, we reported and recommended that subjects with obesity aged 30–44 should be screened every 2 years, while those with a normal body mass index in the same age group would not need screening for the next 10 years because the paces of HbA1c progression are different based on age and BMI [6]. These results considered only HbA1c test characteristics and did not include economic impact in determining the optimal interval for HbA1c screening. People whose screening results were false positive may receive unnecessary treatment, and people whose screening results were false negative may incur higher treatment costs. When optimal screening intervals are introduced based on patient risk stratification, it would be possible to eliminate unnecessary tests as well as to minimize the chance of failing to detect affected patients.

Our study aimed to determine the optimal HbA1c test interval strategy to detect new type 2 diabetes mellitus (T2DM) cases in a healthy population stratified by age and body mass index (BMI), considering HbA1c test characteristics and cost effectiveness.

## Methods

**Fig. 1:**
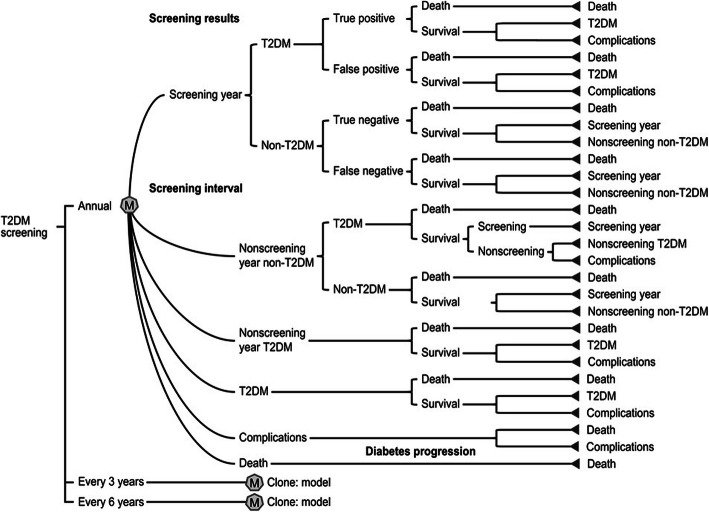
The tree structures in the state transition model of three different strategies for type 2 diabetes screening

We built a state transition model of screening results and type 2 diabetes disease progression to simulate lifetime diabetes-related health care costs and QALYs. Our target population comprised individuals who had no history of T2DM or cardiovascular events. We stratified the population into age categories of 30–44, 45–59 and 60–74 years old. For each age category, we also stratified patients into BMI status as follows: underweight, BMI < 18.5; normal weight, BMI 18.6–24.9; overweight, BMI 25–29.9; and obesity, BMI ≥30 [7, 8]. In total, 12 stratifications were used to create the state transition models. We used the same tree structure for all 12 models with different parameter values. The tree structures in the state transition model comprise three main branches (Fig. 1), “screening results by HbA1c test”, “no screening year” and “T2DM progression”. Screening result branches consist of parameters: incidence of T2DM and sensitivity and specificity of HbA1c testing to determine how many individuals from the population go to the T2DM progression branch. In the no screening year branch, people are classified as the condition that was determined in the screening results branch. For example, if a person was categorized as false negative, they had a higher complication rate while they were in the no screening year because they technically missed a chance for early detection and treatment. In the T2DM progression branch, people die or may experience complications based on each relative risk. A first-order Monte Carlo simulation (microsimulation) of a hypothetical cohort of 50,000 people was performed to estimate the lifetime expected costs and expected QALY. The cycle length of the model was set to 1 year. A willingness-to-pay (WTP) threshold of 50,000 USD per QALY gained was used as the acceptable level for ICER, and an annual discount rate of 2% was used to calculate both costs and benefits, following the current guidelines [9]. The incremental cost-effectiveness ratio (ICER) was estimated as an indicator of cost effectiveness of the test interval using the formula: ICER = (Cost _interval_a_ – Cost _interval_b_)/(QALYd _interval_a_ – QALY _interval_b_). The TreeAge Pro 2016 (TreeAge Software, Williamstown, MA, USA) was used for model construction and analyses.

### Parameters in the state transition models

#### Screening results by HbA1c test

The sensitivity and specificity of the HbA1c test were calculated in each population using real data from St. Luke’s International Hospital, Tokyo. We used the same data set and methodology to calculate sensitivity and specificity when we recommended different intervals based on risk stratification [6] (Table 1). The methodology has been described in detail elsewhere [10, 11]. Briefly, HbA1c was calculated to generate predicted HbA1c by linear random effect models adjusted with gender, age and baseline BMI. Sensitivity and specificity were calculated by comparing the observed HbA1c value and the generated predicted HbA1c value as the gold standard.

**Table 1:** Sensitivity and specificity rates applied to the state transition models

Age group	BMI group	Interval	HbA1c sensitivity (%)	HbA1c specificity (%)
30–44	Underweight (< 18.5 kg/m^2^)	Annual	≒0.0	100.0
		3 years	50.0	100.0
		10 years	81.8	99.9
	Normal (18.5–25 kg/m^2^)	Annual	≒0.0	100.0
		3 years	47.5	99.9
		6 years	68.1	99.9
	Overweight (25–30 kg/m^2^)	Annual	≒0.0	100.0
		3 years	54.5	99.7
		4 years	57.8	99.6
	Obese (< 30 kg/m^2^)	Annual	≒0.0	100.0
		2 years	37.5	99.1
		3 years	62.2	98.6
45–59	Underweight (< 18.5 kg/m^2^)	Annual	≒0.0	100.0
		3 years	66.7	99.8
		10 years	70.0	99.7
	Normal (18.5–25 kg/m^2^)	Annual	≒0.0	100.0
		3 years	56.0	99.7
		6 years	73.5	99.5
	Overweight (25–30 kg/m^2^)	Annual	2.5	100.0
		3 years	61.1	99.1
		4 years	66.8	99.0
	Obese (< 30 kg/m^2^)	Annual	≒0.0	100.0
		3 years	62.0	98.0
		4 years	71.4	98.0
60–74	Underweight (< 18.5 kg/m^2^)	Annual	≒0.0	100.0
		3 years	50.0	99.5
		6 years	87.5	99.3
	Normal (18.5–25 kg/m^2^)	Annual	≒0.0	100.0
		3 years	50.3	99.3
		7 years	72.7	99.1
	Overweight (25–30 kg/m^2^)	Annual	≒0.0	100.0
		3 years	52.3	99.1
		5 years	64.5	98.7
	Obese (< 30 kg/m^2^)	Annual	≒0.0	100.0
		3 years	60.0	97.5
		4 years	70.0	97.5

#### Cost

Direct costs estimated in this study include screening cost and type 2 diabetes treatment. Indirect costs were not considered in this study. We estimated the unit costs of screening with the HbA1c test (including consumables, staff time and laboratory processing costs) as USD 80.00. We had to assume the fee for type 2 diabetes screening with HbA1c because medical cost for prevention is not covered in Japan; thus, there was no official price list for the type 2 diabetes screening test in Japan. We estimated it by summing the cost for a T2DM patient with a stable glycemic condition who received a routine HbA1c test followed by a doctor’s consultation.

For the treatment fee, we used published cost data for T2DM. Fukuda et al. [12] reported detailed treatment costs for T2DM as well as proportion rates for each T2DM-related complication. In our model, we aggregated to one treatment cost for any complication based on the proportions of Japanese people experiencing complications. We also estimated annual treatment fees for false positive patients by summing nutrition education and physical exercise education and assuming no drug prescription fees.

#### Utility

We assumed the utility value to be that of full health and set at 1. We assigned 0.785 utility for those with T2DM without any complications based on a previous review [13]. We calculated a single utility value for those with T2DM with any complication based on multiple studies. Fukuda et al. [12] thoroughly reported treatment costs for patients with T2DM and the proportion of T2DM-related complications using the Japan Medical Data Center Claims Database. We first retrieved utility values for each T2DM-related complication from previous studies and then weighted each utility value based on the proportion reported to aggregate into one utility, which represents the average utility value for patients with T2DM with any complication (Table 2).

**Table 2:** Parameters used in the tree model

	Items	Type of distribution of PSA	Point estimate	Distribution parameters for PSA	Ref
Cost (USD)	Screening cost	Gamma	100	α =27.3, λ =1/292	
	Annual treatment fee among T2DM patients without complication	Gamma	3500	α =84,146, λ =1/3	[12]
	Annual treatment fee among T2DM patients with complication	Gamma	8000	α =26,540, λ =1/22	[12]
	Annual treatment fee among false positive patients with no medication	Gamma	1400	α =1752, λ =1/60	[12]
	Discount rate	–	0.02	–	[14]
Utility	Utility for healthy population	–	1		[13]
	Utility for T2DM patients without complication	–	0.785	–	[13]
	Utility for T2DM patients with complication	–	0.638	–	[13]
Risk	Mortality rate among healthy population	Life Table	–	–	
	Relative risk towards mortality rate among T2DM patients with complications	LogNormal	5.61	Log (mean) = 1.65, SE = 0.08	[15]
	Relative risk towards mortality rate among T2DM patients without complications	LogNormal	2.61	Log (mean) =0.95, SE = 0.14	[15]
	Annual complication rate	Beta	0.014	α =2.27, β =160.3	[16]
	Relative risk towards complication rate among T2DM patients with treatment	LogNormal	0.79	Log (mean) = −0.23, SE = 0.09	[17]

#### Risks from type 2 diabetes

The age-dependent mortality rate for people without type 2 diabetes was obtained from the life table reported by the Ministry of Health, Labour, and Welfare in Japan [18]. We assumed that people with type 2 diabetes receiving the appropriate treatment would achieve the same mortality rate as people without type 2 diabetes based on a recent study [19]. The relative risks of mortality for patients with type 2 diabetes with and without complications were set to 5.22 and 2.61, respectively [15, 20].

The annual incidence of T2DM complications was set to 0.014 based on a previous study [16]. Furthermore, we assumed that patients receiving appropriate treatment would experience fewer complications than those receiving no treatment. There are no published complication rate data for patients with no treatment; thus, we decided to retrieve data from the report, which compared metformin therapy versus conventional therapy. We treated conventional therapy as no treatment, so patients receiving appropriate treatment with metformin therapy had a 0.78-fold lower complication rate [17].

#### Probabilistic sensitivity analysis (PSA)

The robustness of the model results was assessed the model assumptions and parameter uncertainties. For this purpose, deterministic and probabilistic sensitivity analyses (PSA) were used for each parameter shown in Table 2. The PSA explored the uncertainties in the model parameters by randomly sampling 1000 people with 1000 iterations on each parameter distribution. We calculated the cost, QALYs and ICERs from this sample.

## Results

Table 3 shows each QALY, incremental QALY, cost, incremental cost and ICER for 12 stratified groups based on age and BMI level. The HbA1c test interval to detect T2DM in a healthy population varies by age and BMI. Three-year intervals were the most cost effective in obesity at all ages —30-44: $15,034/QALY, 45–59: $11,849/QALY, 60–74: $8685/QALY—compared with the other two interval strategies. The three-year interval was also the most cost effective in the 60–74-year-old groups—underweight: $11,377/QALY, normal: $18,123/QALY, overweight: $12,537/QALY—and overweight in the 45–59-year-old group; dominant. In other groups, the screening interval for detecting T2DM was found to be longer than 3 years, as previously reported. Annual screenings dominated in many groups with low BMI and in younger age groups. Suggested screening strategies for all groups are shown in the Table 4. According to PSA, the results were consistent with the basic analysis (Additional file 1). Based on the probability distribution of ICER, the QALY does not show much difference among any groups.

**Table 3:** Results for the cost effectiveness analysis (Monte Carlo micro-simulation 50,000 times)

Population		Interval	QALY	Incremental QALY	Cost ($)	Incremental cost ($)	ICER ($/QALY)
Age	BMI group						
30–44	Underweight	Annual	30.12	–	2467.16	–	Dominated
		3-year	30.16	0.01	963.10	557.19	93,898.79 (vs 10-year)
		10-year	30.16	–	405.92	–	–
	Normal	Annual	30.13	–	2489.28	–	Dominated
		3-year	30.15	0.01	1112.80	385.93	63,295.18 (vs 6-year)
		6-year	30.14	–	726.86	–	–
	Overweight	Annual	29.86	–	2894.63	–	Dominated
		3-year	29.97	–	2678.14	–	Dominated
		4-year	29.97	–	2477.31	–	Dominant
	Obese	Annual	28.88	–	4313.80	–	–
		2-year	29.18	–	8965.05	–	Dominated
		3-year	29.19	0.31	8948.87	4635.07	15,034.02 (vs Annual)
45–59	Underweight	Annual	23.54	–	1949.99	–	Dominated
		3-year	23.55	–	879.01	–	Dominated
		10-year	23.55	–	408.86	–	Dominant
	Normal	Annual	23.45	–	2081.91	–	Dominated
		3-year	23.48	–	1445.89	–	Dominated
		6-year	23.49	–	1107.16	–	Dominant
	Overweight	Annual	23.17	–	2763.11	–	Dominated
		3-year	23.20	–	2753.06	–	Dominated
		4-year	23.26	–	2908.66	–	–
	Obese	Annual	22.27	–	3024.01	–	–
		3-year	22.66	0.4	7647.58	4623.57	11,849.71 (vs Annual)
		4-year	22.63	–	7348.82	–	Dominated
60–74	Underweight	Annual	15.74	–	1325.15	–	Dominated
		3-year	15.77	0.02	651.84	198.73	11,377.06 (vs 6-year)
		6-year	15.75	–	453.10	–	–
	Normal	Annual	15.69	–	1379.50	–	Dominated
		3-year	15.72	0.02	1039.23	319.93	18,123.44 (vs 7-year)
		7-year	15.70	–	719.29	–	–
	Overweight	Annual	15.47	–	1481.22	–	–
		3-year	15.56	0.02	1829.22	275.64	12,537.81 (vs 5-year)
		5-year	15.54	0.07	1553.58	72.36	1006.48 (vs Annual)
	Obese	Annual	15.01	–	1617.53	–	–
		3-year	15.23	0.22	3552.88	1935.34	8685.76 (vs Annual)
		4-year	15.21	–	3363.21	–	Dominated

Underlined intervals are found to be the most cost-effective strategies with willingness-to-pay threshold of $50,000. Units of costs, incremental costs and ICER is in USD$

**Table 4:** Suggested screening strategies based on cost-effectiveness analysis, stratified by age and BMI group

		Age group		
		30–44	45–59	60–74
BMI group	Underweight	10-year	10-year	3-year
	Normal	6-year	6-year	3-year
	Overweight	4-year	3-year	3-year
	Obese	3-year	3-year	3-year

## Discussion

According to our cost-effectiveness analysis results, T2DM screening programs for healthy populations should consider risk stratification for T2DM. The optimal interval seems to vary from 3 to 10 years, and some groups do not require the 3-year screening interval that current guidelines suggest. There were no groups that warrant annual screening, which is required by Japanese law.

As we reported previously, HbA1c progression in the high-risk group was remarkable, while HbA1c stayed at the same level or plateaued in the low-risk group. HbA1c progression in the lower risk group in the short interval is often affected by noise, which is originally possessed by HbA1c [6]. When deciding the appropriate screening testing frequencies, we should consider how well a screening program can distinguish true type 2 diabetes patients from true nondiabetic patients with the lowest financial burden to public health. To achieve this, health policies need to simultaneously integrate all influences according to patients’ risk factors, HbA1c test characteristics and financial impact.

Annual intervals dominated for all age groups of underweight and normal BMI individuals and the 30–44-year-old overweight group. In other groups, annual intervals did not dominated; however, annual intervals were not warranted based on the ICER.

A 3–5 year monitoring interval suggested by Kahn et al. [1] while providing good evidence for cost effectiveness, does not apply to those over 45 years old. Chen et al. [21] concluded that a 5-year interval for all age groups would be the most cost-effective strategy. Interestingly, Hoerger et al. [22] concluded that the most cost-effective strategy is targeted screening of 55- to 75-year-old patients with hypertension. Moreover, Brateanu et al. [5] suggested that the optimal interval for type 2 diabetes screening should be decided by patients’ risk score regarding the cause of type 2 diabetes; they concluded that patients in the highest risk group could be rescreened after 8 months, while those in the intermediate and lowest risk categories could be rescreened between 3 and 5 years. Our study also supports that risk stratification should be considered when deciding the optimal interval for type 2 diabetes. To the best of our knowledge, this study is the first to investigate the best interval for T2DM screening, considering both test characteristics and cost effectiveness. We have previously suggested optimal HbA1c test screening results based on risk stratification of patients and test characteristics of the HbA1c test. This time, we found that the results could change when we integrated economic evaluation as well as patient risk stratification and HbA1c test characteristics. Patient risk stratification, HbA1c test characteristics and economic impact on public health not only should be used as the basis of health policy but also should be integrated and evaluated to maximize the effectiveness of mass screening.

As in many other studies, this study also includes uncertainties in our models. All patients will receive treatment once the screening test results are positive. We estimated that patients with T2DM will live if appropriate treatment is provided based on a previous report. In the real world, there should be a certain number of patients who do not visit clinics for treatment even after they receive positive results by screening tests. We estimated a lower complication rate if patients received treatment (true positive) and a higher complication and mortality rate if patients did not receive treatment (false negative). It was impossible to obtain published data for patients who did not receive treatment; thus, we had to retrieve data from the report comparing new treatment regimens versus conventional treatment regimens [17]. The intention of our study is not to identify the best risk stratification strategy. We stratified patients by age and current BMI level; however, there is room for consideration that BMI level in childhood should be applied instead of current BMI level, which has been reported to be more important for predicting the cause of new type 2 diabetes in adulthood [23]. We also did not consider due to data inaccessibility data for family history and patient medical history such as presence of prediabetes, hyperlipidemia and hypertension, which could be the candidate factors for robust stratification.

## Conclusions

Annual screening to detect T2DM was not cost effective and should not be used for any population. The three-year screening interval was optimal for all elderly populations, the obesity at all ages and the overweight 45–59-year-old group. Among low BMI and younger age groups, the optimal HbA1c test interval can be longer than 3 years.

## Supplementary Information

**Additional file 1: Table S1.** Results of the Probabilistic Sensitivity Analysis-1000 individuals with 1000 Monte Carlo Simulations. **Figure S1.** Age 30-44 years old: Outcomes of 1000 simulations from the societal perspective. **Figure S2.** Age 45-59 years old: Outcomes of 1000 simulations from the societal perspective. **Figure S3.** Age 60-74 years old: Outcomes of 1000 simulations from the societal perspective. **Figure S4.** Age 30-44 years old: Monte Carlo Simulation % cost effectiveness strategies. **Figure S5.** Age 45-59 years old: Monte Carlo Simulation % cost effectiveness strategies. **Figure S6.** Age 60-74 years old: Monte Carlo Simulation % cost effectiveness strategies.

## Data Availability

All parameters used in the state transition models are presented on Tables 2 and 1. Original files of each state transition models are available upon request.

## Abbreviations

BMI: Body mass index
T2DM: Type 2 diabetes mellitus
HbA1c: Hemoglobin A1c
