# Supplementary material for: Cost-effectiveness analysis for HbA1c test intervals to screen patients with type 2 diabetes based on risk stratification
Source: BMC Endocr Disord. 2021 May 22;21:105. doi: 10.1186/s12902-021-00771-0 (PMC8141129; doi:10.1186/s12902-021-00771-0)
Supplement: Supplementary file 1 — Additional file 1: Table S1. Results of the Probabilistic Sensitivity Analysis-1000 individuals with 1000 Monte Carlo Simulations. Figure S1. Age 30-44 years old: Outcomes of 1000 simulations from the societal perspective. Figure S2. Age 45-59 years old: Outcomes of 1000 simulations from the societal perspective. Figure S3. Age 60-74 years old: Outcomes of 1000 simulations from the societal perspective. Figure S4. Age 30-44 years old: Monte Carlo Simulation % cost effectiveness strategies. Figure S5. Age 45-59 years old: Monte Carlo Simulation % cost effectiveness strategies. Figure S6. Age 60-74 years old: Monte Carlo Simulation % cost effectiveness strategies. [file 12902_2021_771_MOESM1_ESM.docx]

**Appendix Table 1.**

Results of the Probabilistic Sensitivity Analysis-1,000 individuals with 1,000 Monte Carlo Simulations

| Population | | Interval | QALY | Incremental QALY | Cost ($) | Incremental cost ($) | ICER ($/QALY) |
| --- | --- | --- | --- | --- | --- | --- | --- |
| Age | BMI group |  |  |  |  |  |  |
| 30-44 | Underweight | Annual | 30.15 | ― | 2,464.40 | ― | Dominated |
|  |  | 3-year | 30.15 | ― | 976.09 | ― | Dominated |
|  |  | 10-year | 30.16 | ― | 426.58 | ― | Dominant |
|  | Normal | Annual | 30.13 | ― | 2,495.39 | ― | Dominated |
|  |  | 3-year | 30.14 | 0.002 | 1,117.75 | 395.94 | 181,321.36  (vs 6-year) |
|  |  | 6-year | 30.14 | ― | 721.81 | ― | ― |
|  | Overweight | Annual | 29.87 | ― | 2,873.92 | ― | Dominated |
|  |  | 3-year | 29.94 | ― | 2,720.52 | ― | Dominated |
|  |  | 4-year | 29.94 | ― | 2,466.16 | ― | Dominant |
|  | Obese | Annual | 28.88 | ― | 4,360.47 | ― | ― |
|  |  | 2-year | 29.18 | ― | 9,013.37 | ― | Dominated |
|  |  | 3-year | 29.21 | 0.33 | 9,014.82 | 4,654.30 | 14,035.31  (vs Annual) |
| 45-59 | Underweight | Annual | 23.56 | ― | 195,290.60 | ― | Dominated |
|  |  | 3-year | 23.57 | 0.003 | 86,842.28 | 459.46 | 182,263.40  (vs 10-year) |
|  |  | 10-year | 23.56 | ― | 40,896.47 | ― | ― |
|  | Normal | Annual | 23.42 | ― | 2,055.86 | ― | Dominated |
|  |  | 3-year | 23.46 | ― | 1,491.09 | ― | Dominated |
|  |  | 6-year | 23.46 | ― | 1,137.62 | ― | Dominant |
|  | Overweight | Annual | 23.16 | ― | 2,790.12 | ― | ― |
|  |  | 3-year | 23.26 | 0.01 | 3,098.03 | 235.80 | 26,771.81  (vs 4-year) |
|  |  | 4-year | 23.25 | 0.09 | 2,862.22 | 72.10 | 802.17  (vs Annual) |
|  | Obese | Annual | 22.27 | ― | 3,068.62 | ― | ― |
|  |  | 3-year | 22.67 | 0.01 | 7,555.49 | 262.53 | 28,842.73  (vs 4-year) |
|  |  | 4-year | 22.66 | 0.38 | 7,292.96 | 4,224.34 | 11,000.99  (vs Annual) |
| 60-74 | Underweight | Annual | 15.75 | ― | 1330.28 | ― | Dominated |
|  |  | 3-year | 15.76 | 0.004 | 661.71 | 194.53 | 47,742.19  (vs 6-year) |
|  |  | 6-year | 15.76 | ― | 467.18 | ― | ― |
|  | Normal | Annual | 15.65 | ― | 1366.64 | ― | Dominated |
|  |  | 3-year | 15.70 |  | 1008.83 | 294.71 | 44026.83  (vs 7-year) |
|  |  | 7-year | 15.69 | ― | 714.11 | ― | ― |
|  | Overweight | Annual | 15.45 | ― | 1,453.632 | ― | ― |
|  |  | 3-year | 15.54 | 0.01 | 1,810.007 | 246.61 | 30,620.69  (vs 5-year) |
|  |  | 5-year | 15.53 | 0.08 | 1,563.397 | 109.7649 | 1,347.50  (vs Annual) |
|  | Obese | Annual | 15.02 | ― | 1,629.16 | ― | ― |
|  |  | 3-year | 15.25 | 0.004 | 3,606.67 | 176.59 | 40,588.01  (vs 4-year) |
|  |  | 4-year | 15.25 | 0.22 | 3,430.09 | 1,800.93 | 8,108.36  (vs annual) |

Underlined intervals are found to be the most cost effective strategies with a willingness-to-pay threshold of $50,000. Units of costs, incremental costs and ICER is in USD$.

Appendix Figure 1: Age 30-44 years old: Outcomes of 1000 simulations from the societal perspective

| Underweight | | 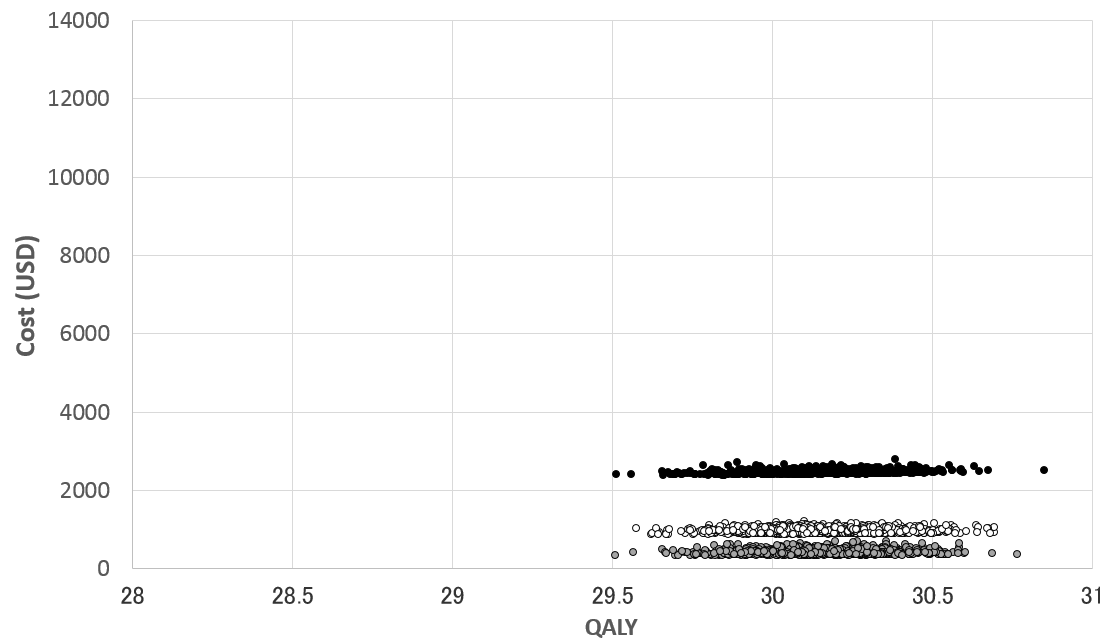 |
| --- | --- | --- |
| ○ | Annual interval |  |
| ● | Every 3 years |  |
| ○ | Every 10 years |  |
|  | |  |
| Normal | | 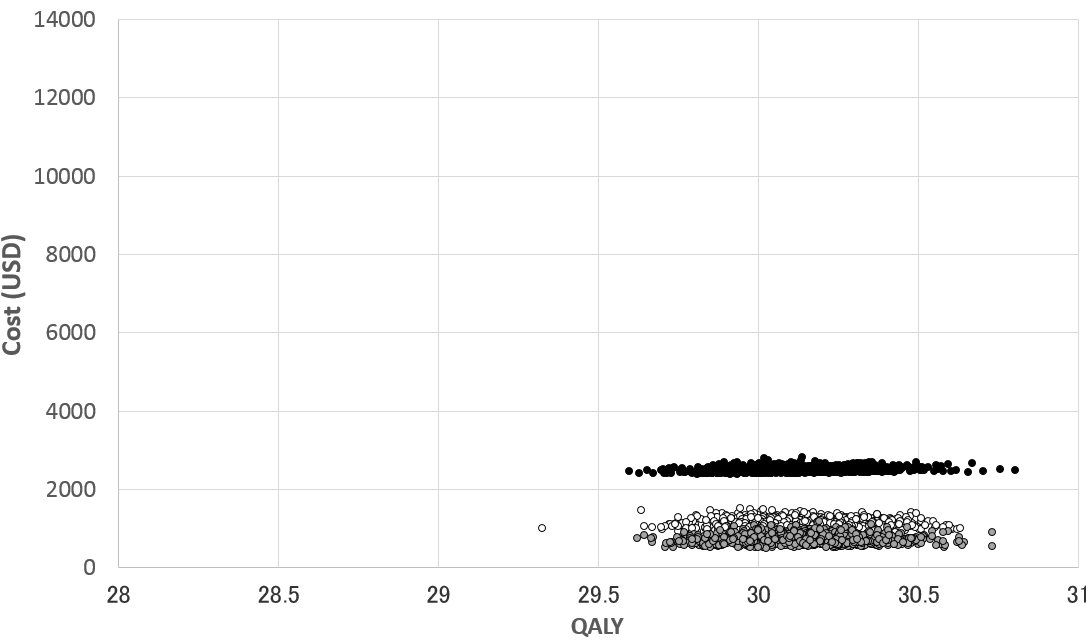 |
| ○ | Annual interval |  |
| ● | Every 3 years |  |
| ○ | Every 6 years |  |
|  | |  |
| Overweight | | 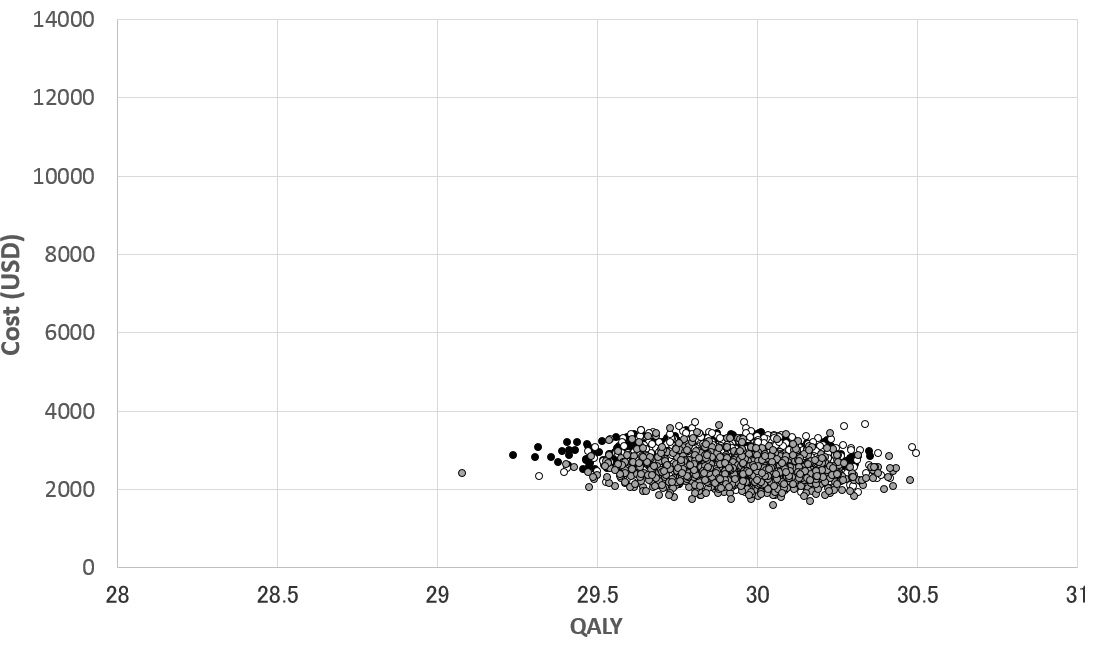 |
| ○ | Annual interval |  |
| ● | Every 3 years |  |
| ○ | Every 6 years |  |
|  | |  |
| Obese | | 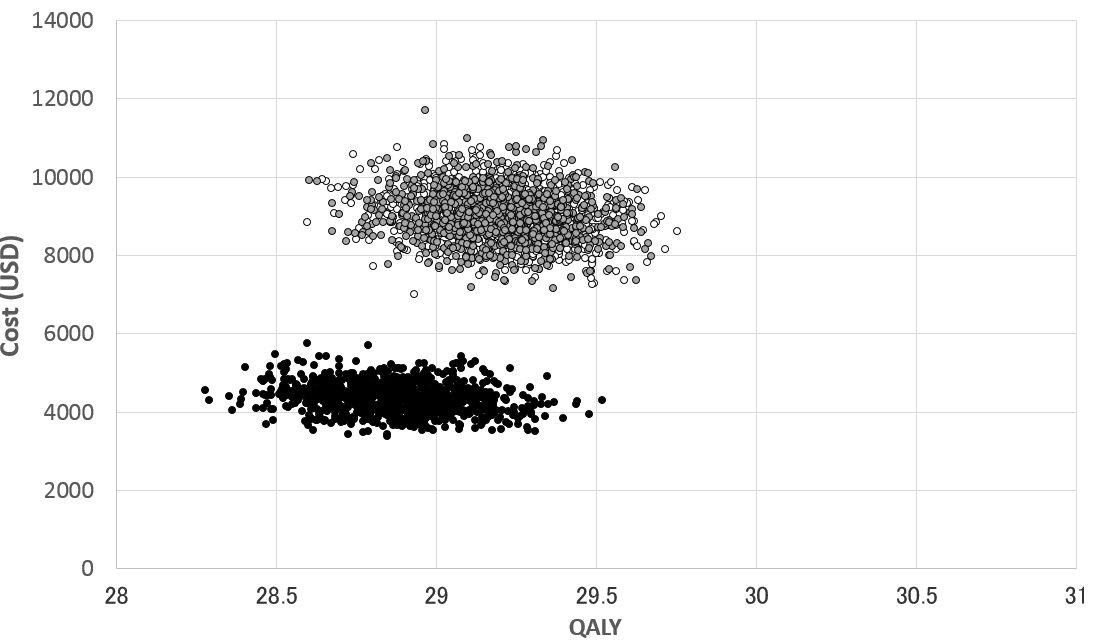 |
| ○ | Annual interval |  |
| ● | Every 3 years |  |
| ○ | Every 2 years |  |
|  | |  |

Appendix Figure 2: Age 45-59 years old: Outcomes of 1000 simulations from the societal perspective

| Underweight | | 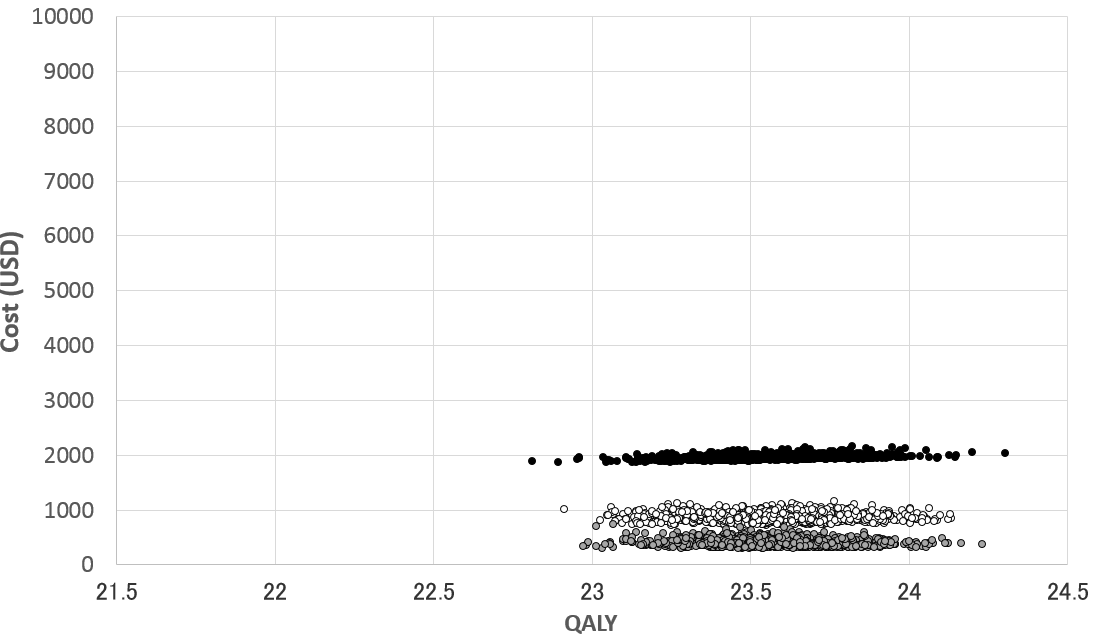 |
| --- | --- | --- |
| ○ | Annual interval |  |
| ● | Every 3 years |  |
| ○ | Every 10 years |  |
|  | |  |
| Normal | | 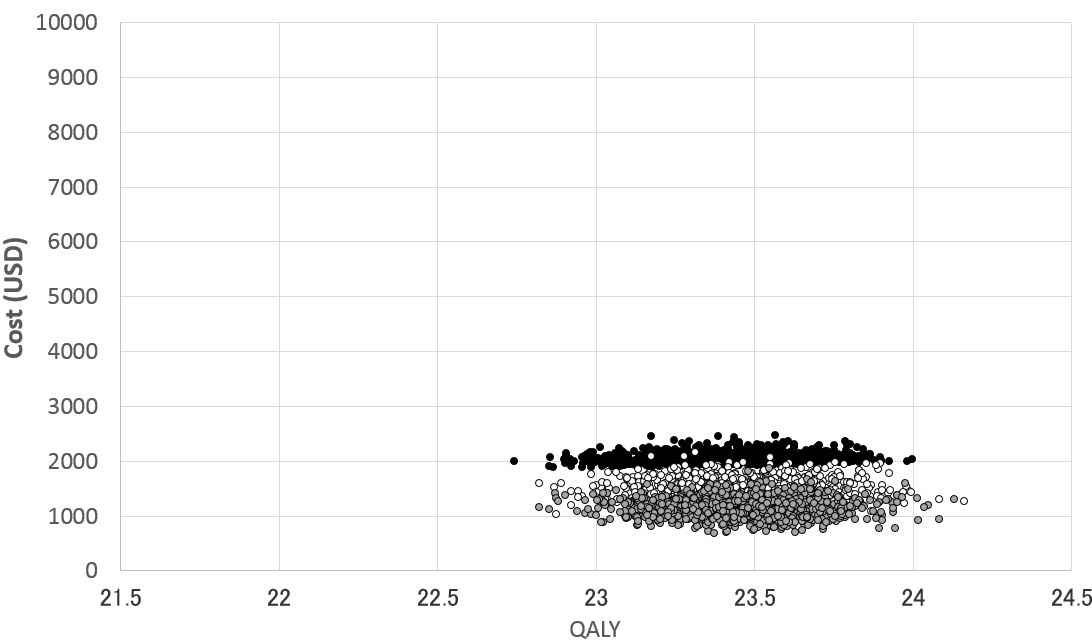 |
| ○ | Annual interval |  |
| ● | Every 3 years |  |
| ○ | Every 6 years |  |
|  | |  |
| Overweight | | 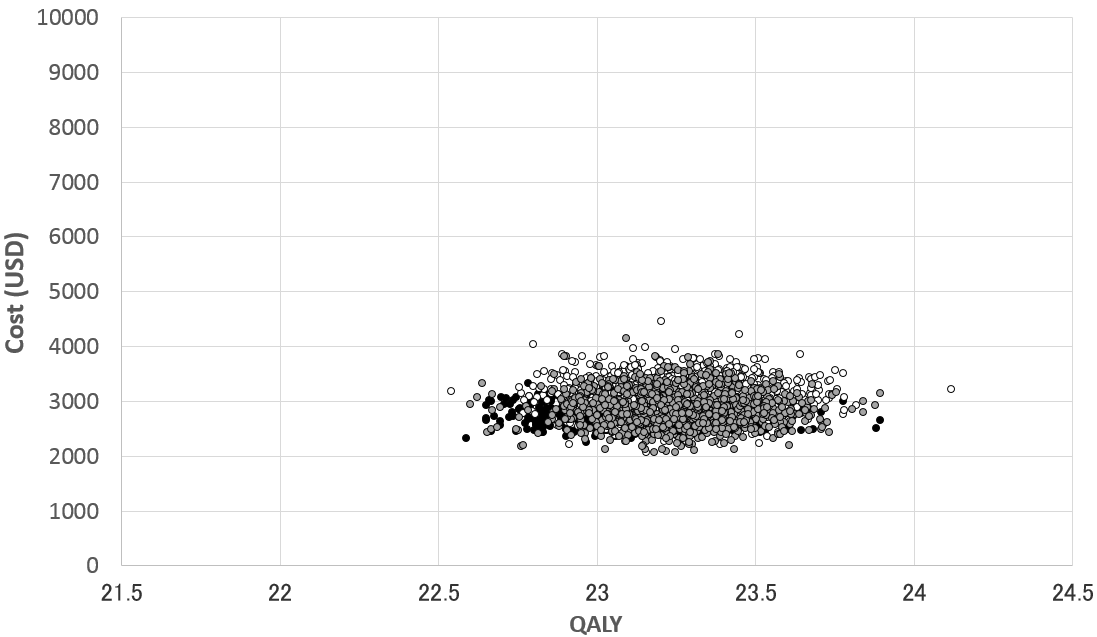 |
| ○ | Annual interval |  |
| ● | Every 3 years |  |
| ○ | Every 4 years |  |
|  | |  |
| Obese | | 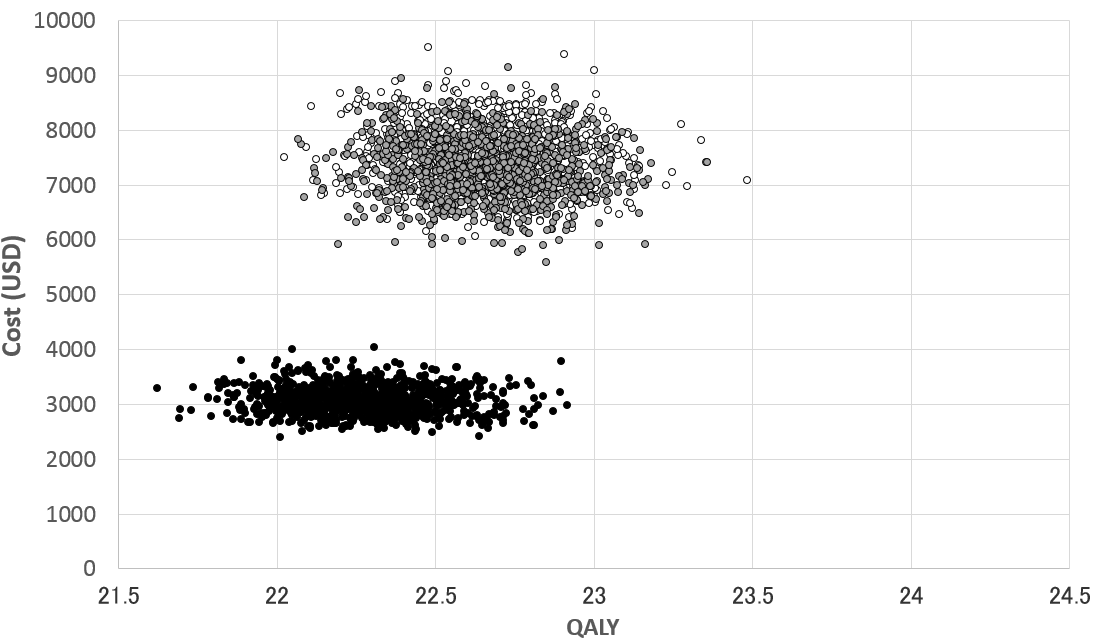 |
| ○ | Annual interval |  |
| ● | Every 3 years |  |
| ○ | Every 4 years |  |
|  | |  |

Appendix Figure 3: Age 60-74 years old: Outcomes of 1000 simulations from the societal perspective

| Underweight | | 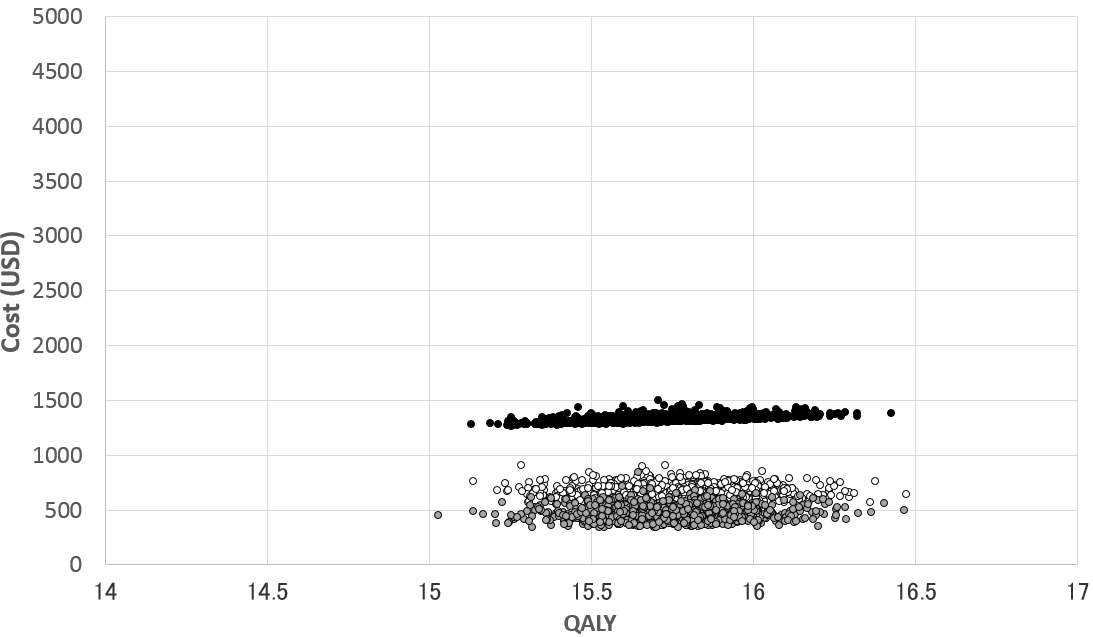 |
| --- | --- | --- |
| ○ | Annual interval |  |
| ● | Every 3 years |  |
| ○ | Every 6 years |  |
|  | |  |
| Normal | | 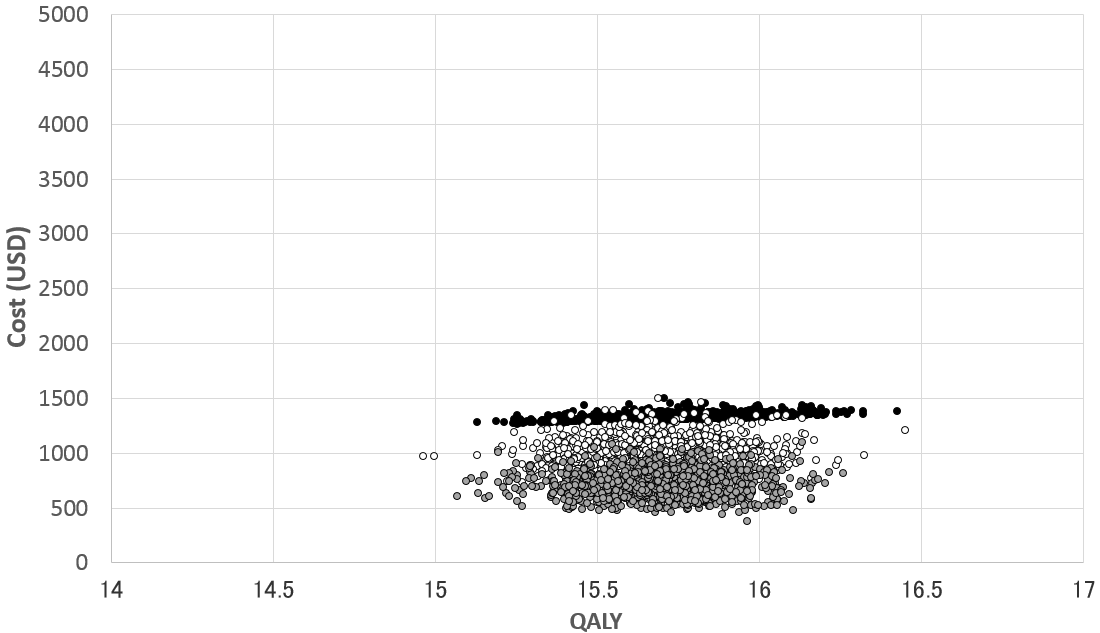 |
| ○ | Annual interval |  |
| ● | Every 3 years |  |
| ○ | Every 7 years |  |
|  | |  |
| Overweight | | 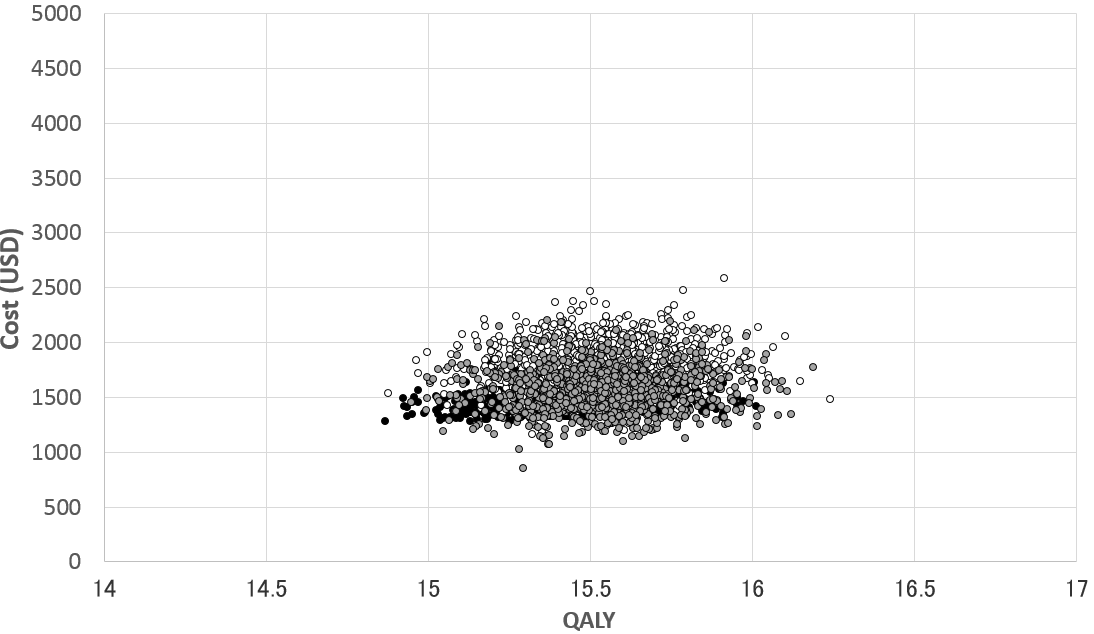 |
| ○ | Annual interval |  |
| ● | Every 3 years |  |
| ○ | Every 5 years |  |
|  | |  |
| Obese | | 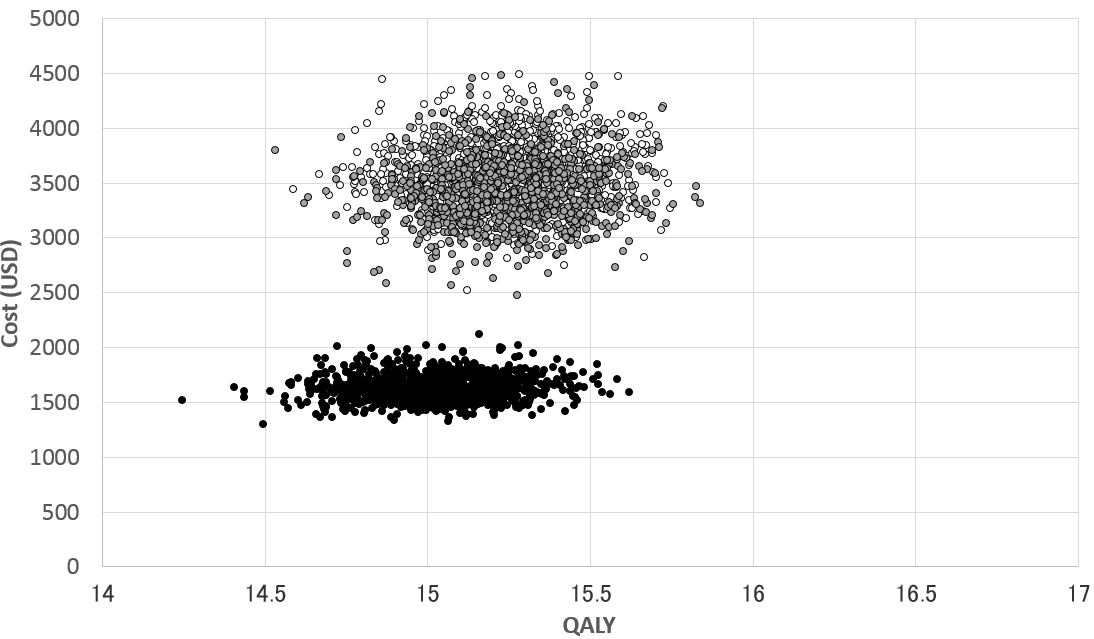 |
| ○ | Annual interval |  |
| ● | Every 3 years |  |
| ○ | Every 4 years |  |
|  | |  |

Appendix Figure 4: Age 30-44 years old: Monte Carlo Simulation % cost effectiveness strategies

| Underweight | | 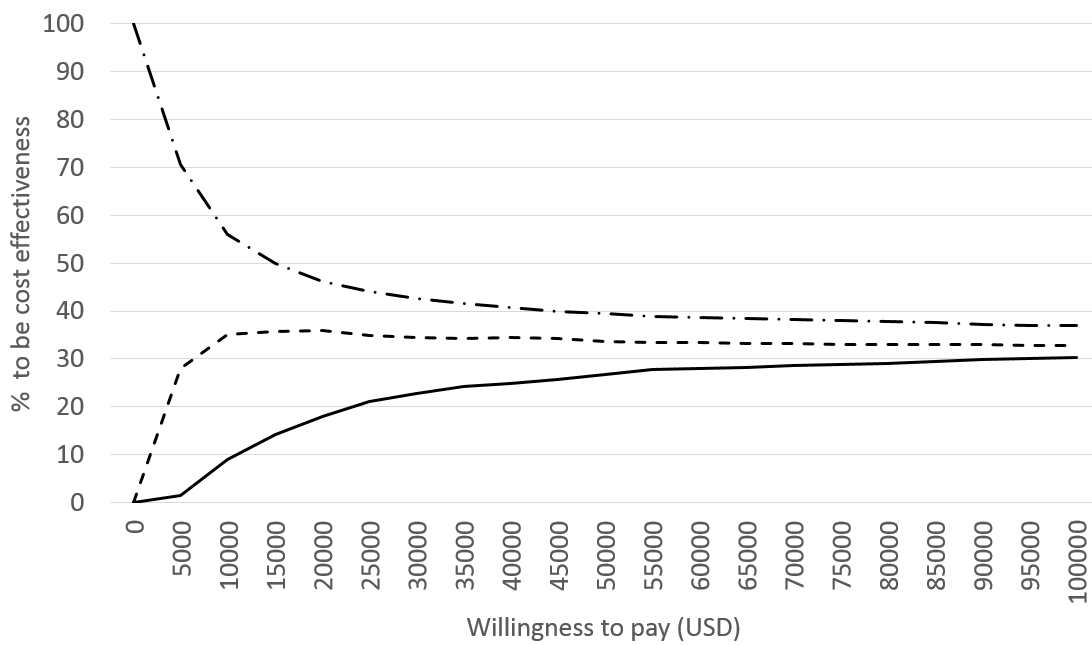 |
| --- | --- | --- |
|  | Annual interval |  |
|  |  |  |
|  | Every 3 years |  |
|  |  |  |
|  | Every 10 years |  |
|  |  |  |
|  | |  |
| Normal | | 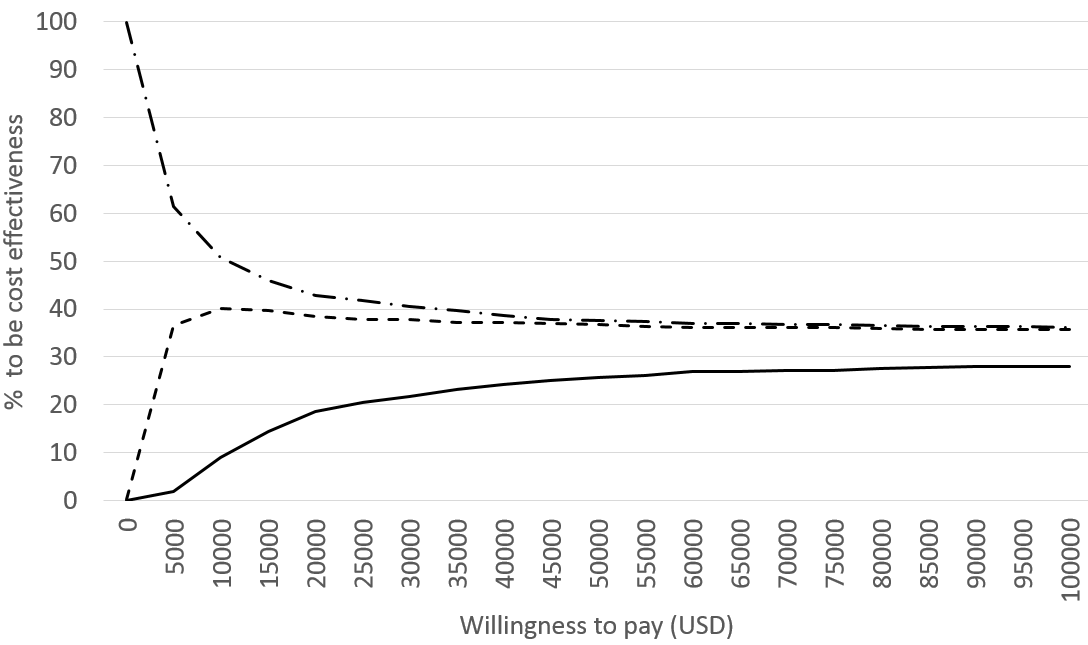 |
|  | Annual interval |  |
|  |  |  |
|  | Every 3 years |  |
|  |  |  |
|  | Every 6 years |  |
|  |  |  |
|  | |  |
| Overweight | | 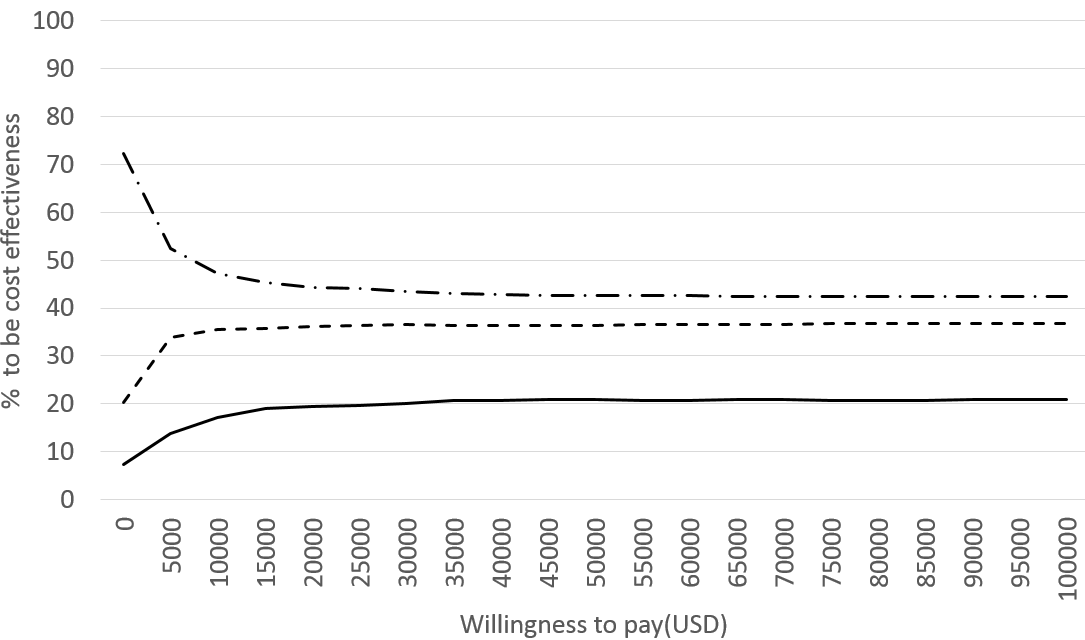 |
|  | Annual interval |  |
|  |  |  |
|  | Every 3 years |  |
|  |  |  |
|  | Every 6 years |  |
|  |  |  |
|  | |  |
| Obese | | 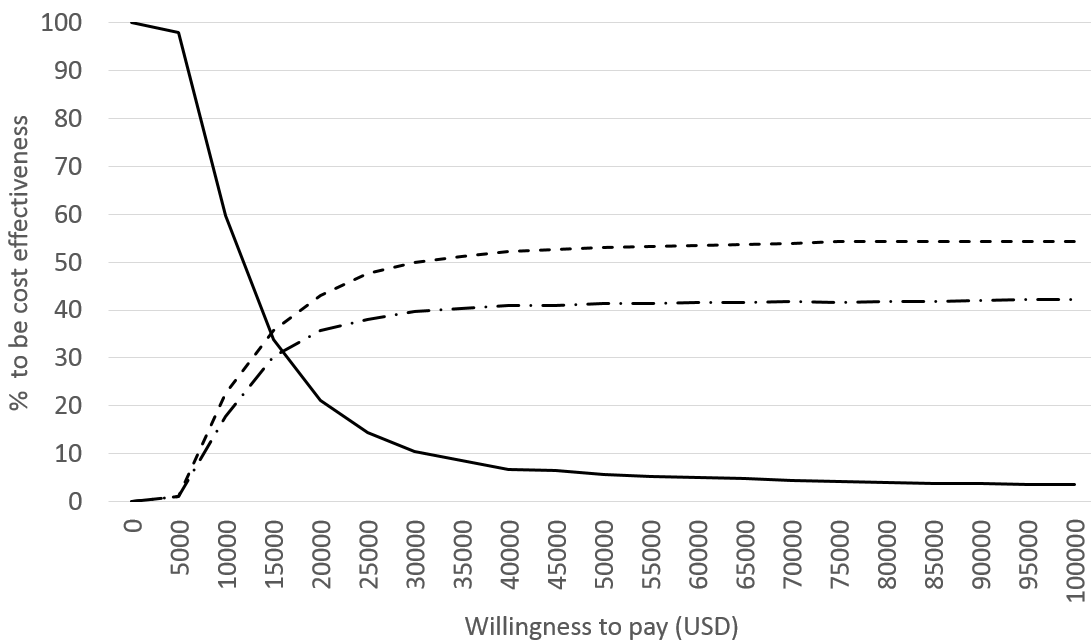 |
|  | Annual interval |  |
|  |  |  |
|  | Every 3 years |  |
|  |  |  |
|  | Every 2 years |  |
|  |  |  |
|  | |  |

Appendix Figure 5: Age 45-59 years old: Monte Carlo Simulation % cost effectiveness strategies

| Underweight | | 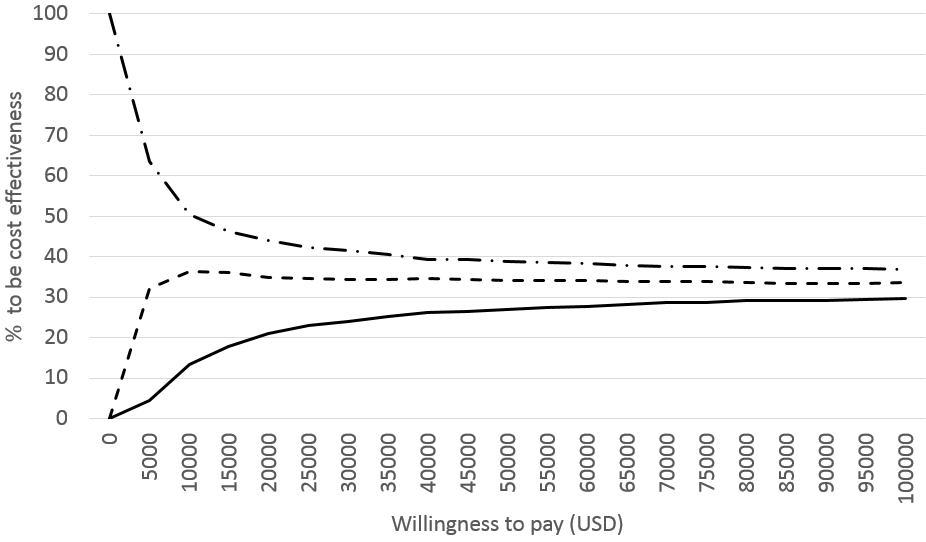 |
| --- | --- | --- |
|  | Annual interval |  |
|  |  |  |
|  | Every 3 years |  |
|  |  |  |
|  | Every 10 years |  |
|  |  |  |
|  | |  |
| Normal | | 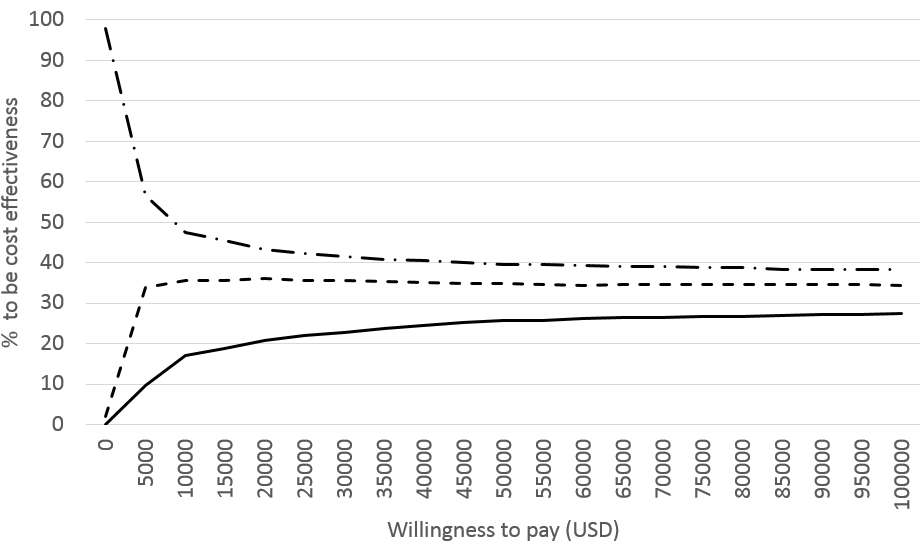 |
|  | Annual interval |  |
|  |  |  |
|  | Every 3 years |  |
|  |  |  |
|  | Every 6 years |  |
|  |  |  |
|  | |  |
| Overweight | | 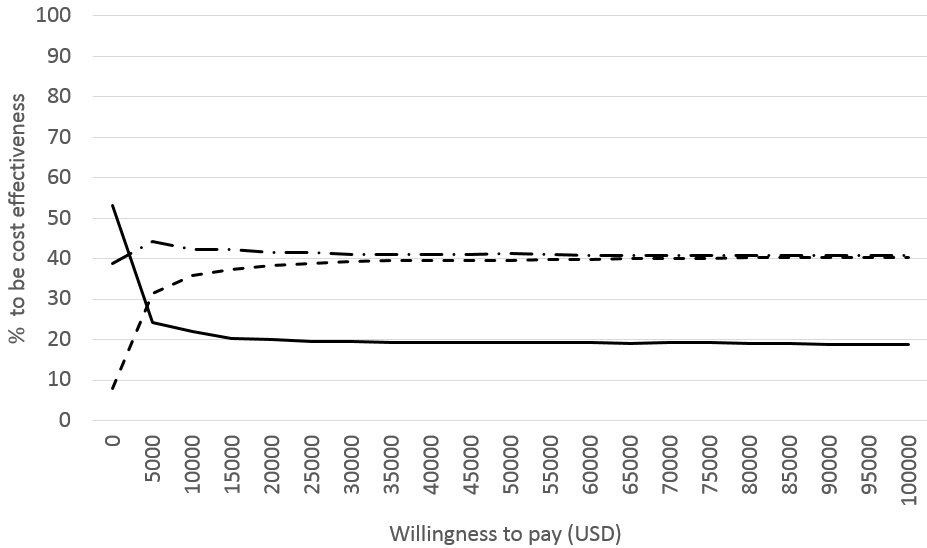 |
|  | Annual interval |  |
|  |  |  |
|  | Every 3 years |  |
|  |  |  |
|  | Every 4 years |  |
|  |  |  |
|  | |  |
| Obese | | 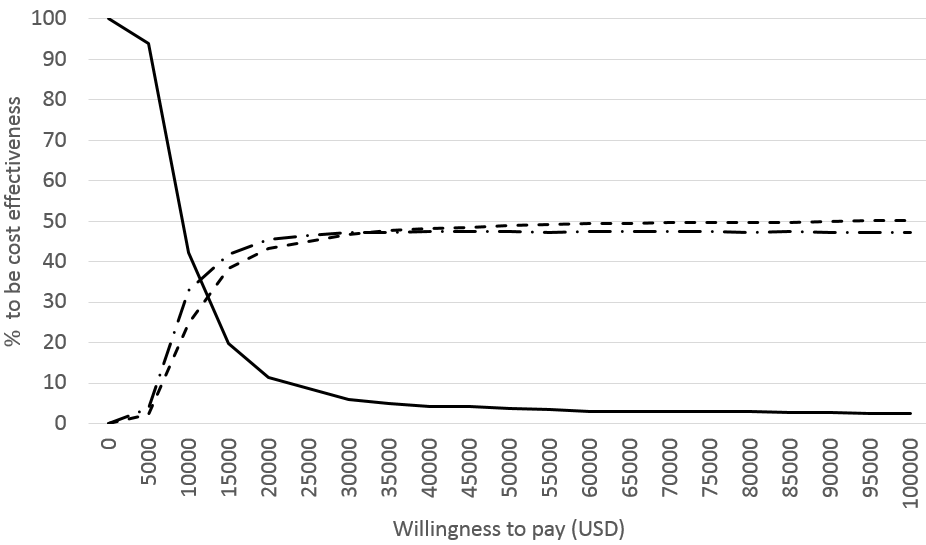 |
|  | Annual interval |  |
|  |  |  |
|  | Every 3 years |  |
|  |  |  |
|  | Every 4 years |  |
|  |  |  |
|  | |  |

Appendix Figure 6: Age 60-74 years old: Monte Carlo Simulation % cost effectiveness strategies

| Underweight | | 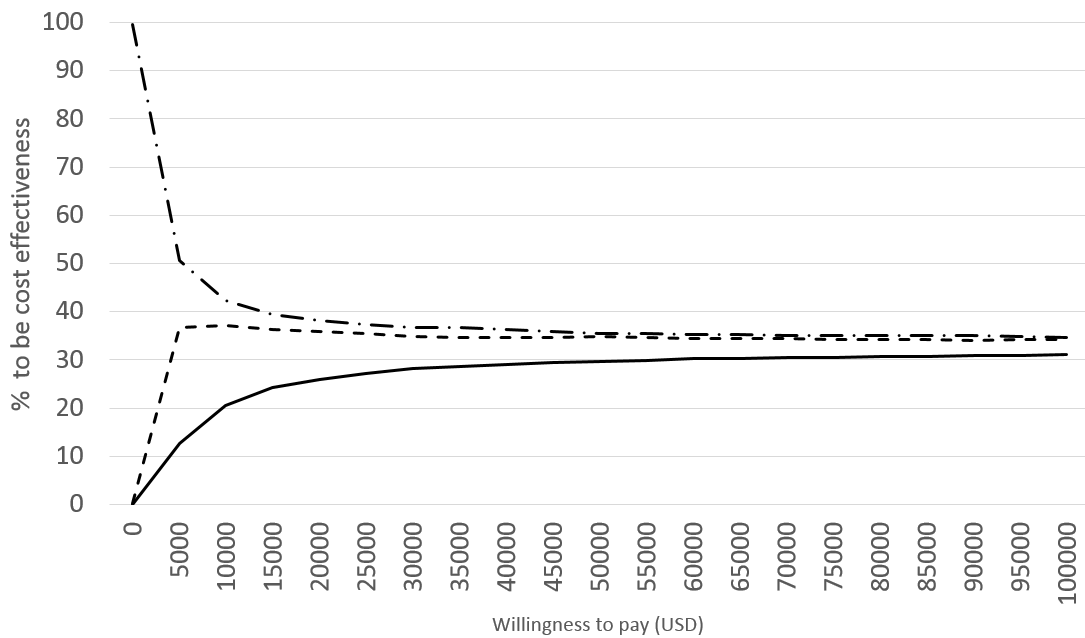 |
| --- | --- | --- |
|  | Annual interval |  |
|  |  |  |
|  | Every 3 years |  |
|  |  |  |
|  | Every 6 years |  |
|  |  |  |
|  | |  |
| Normal | | 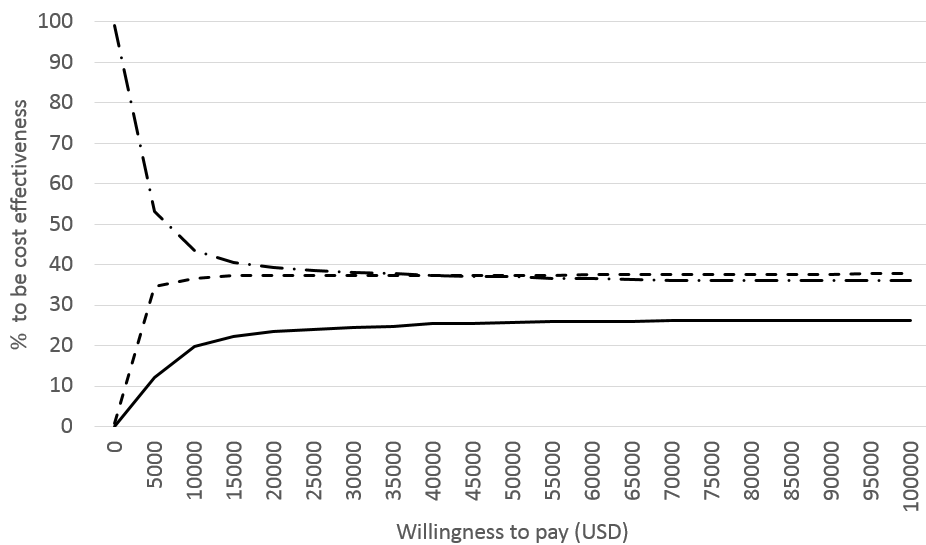 |
|  | Annual interval |  |
|  |  |  |
|  | Every 3 years |  |
|  |  |  |
|  | Every 7 years |  |
|  |  |  |
|  | |  |
| Overweight | | 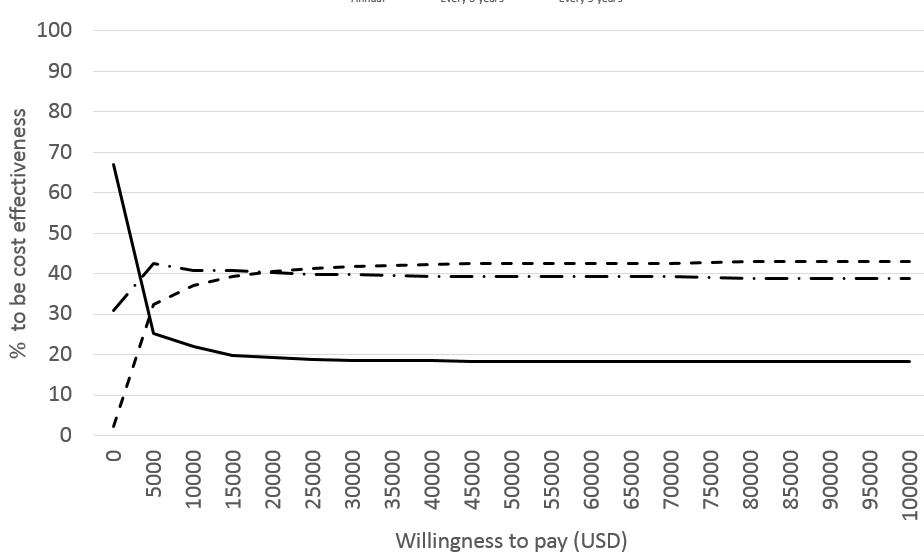 |
|  | Annual interval |  |
|  |  |  |
|  | Every 3 years |  |
|  |  |  |
|  | Every 5 years |  |
|  |  |  |
|  | |  |
| Obese | | 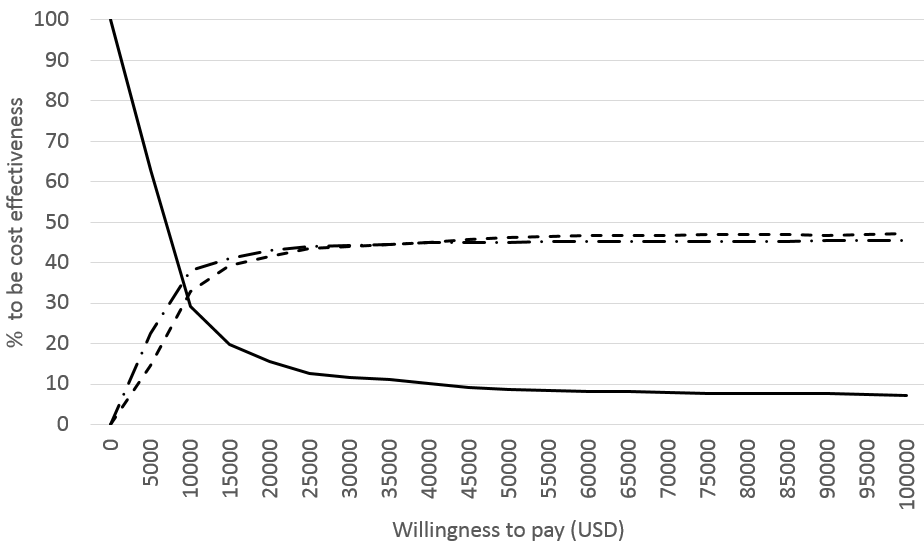 |
|  | Annual interval |  |
|  |  |  |
|  | Every 3 years |  |
|  |  |  |
|  | Every 4 years |  |
|  |  |  |
|  | |  |
